# PRRSV suppresses ER-phagy through Nsp2- and Nsp5-mediated degradation of FAM134B

**DOI:** 10.3389/fcimb.2026.1905056

**Published:** 2026-08-05

**Authors:** Jing Wang, Huiqin Sun, Yue Liang, Dan Luo, Rui Li, Guangxu Xing, Songlin Qiao, Xin-xin Chen

**Affiliations:** 1 Institute for Animal Health, Henan Academy of Agricultural Sciences, Key Laboratory of Animal Immunology of the Ministry of Agriculture, Zhengzhou, China; 2 Longhu Laboratory, Zhengzhou, China; 3 College of Veterinary Medicine, Northwest A&F University, Yangling, Shanxi, China; 4 Shanghai Key Laboratory of Veterinary Biotechnology, School of Agriculture and Biology, Shanghai Jiao Tong University, Shanghai, China; 5 Jiangsu Co-Innovation Center for the Prevention and Control of Important Animal Infectious Diseases and Zoonoses, Yangzhou, China

**Keywords:** ER-phagy, FAM134B, Nsp2, Nsp5, PRRSV

## Abstract

**Background:**

Porcine reproductive and respiratory syndrome virus (PRRSV) is a significant pathogen in the swine industry that causes reproductive failure in sows and respiratory distress in pigs of all ages, leading to substantial economic losses globally. PRRSV manipulates host cellular processes, particularly those associated with endoplasmic reticulum (ER) function. ER-phagy plays a crucial role in maintaining ER homeostasis and enabling cellular adaptations to stress. Whether and how PRRSV modulates ER-phagy remains incompletely understood.

**Methods:**

ER-phagy was monitored by western blotting for free mCherry from the mCherry-Sec61B reporter. FAM134B mRNA and protein levels were examined by RT-qPCR and western blotting, respectively. All 12 PRRSV Nsps were screened for FAM134B-suppressing activity by co-transfection, followed by western blotting. Co-immunoprecipitation (Co-IP) was performed to assess interactions between candidate Nsps and FAM134B, as well as their impact on FAM134B- microtubule-associated proteins light chain 3 (LC3) binding. Viral replication was evaluated by RT-qPCR targeting ORF7 and TCID50 assays.

**Results:**

We investigated the interplay between PRRSV and ER-phagy and discovered that PRRSV suppresses ER-phagy during the late stages of infection. Further analysis revealed that PRRSV employs its Nsps to inhibit the expression of FAM134B. Specifically, PRRSV Nsp2 and Nsp5 interact with FAM134B, promote its degradation and disrupt its binding to microtubule-LC3, thereby impairing ER-phagy.

**Conclusions:**

Collectively, our findings uncover a novel viral strategy to subvert host ER-phagy and provide new insights into PRRSV pathogenesis.

## 1 Introduction

Porcine reproductive and respiratory syndrome (PRRS) is characterized by reproductive disorders in sows and respiratory diseases in pigs of all ages and remains a significant threat to the swine industry worldwide (Albina, 1997; Baron et al., 1992; Butler et al., 2014; Pejsak et al., 1997). Since 2006, highly pathogenic PRRS (HP-PRRS) has emerged and spread widely (Tian et al., 2007; Zhou et al., 2009; Zhou and Yang, 2010), causing severe economic losses to the swine industry in China. PRRSV encodes 8 structural proteins and at least 16 non-structural proteins (Nsps), including Nsp1α, Nsp1β, Nsp2-6, Nsp2TF, Nsp2N, Nsp7α, Nsp7β, and Nsp8-12 (Fang and Snijder, 2010; Snijder et al., 2013).

Autophagy is a highly conserved cellular process responsible for the degradation and recycling of cellular components, thereby maintaining cellular homeostasis (Nakatogawa, 2020). Numerous studies have confirmed that PRRSV triggers autophagy, and this process is associated with viral replication and host immune regulation (Chen et al., 2012; Liu et al., 2012; Sun et al., 2012; Zhou et al., 2023). Based on substrate specificity, autophagy is classified into non-selective autophagy and selective autophagy. Selective autophagy delivers specific substrates to the autophagosome for degradation through the interaction between autophagy receptors and microtubule-associated proteins light chain 3 (LC3) (Birgisdottir et al., 2013). Several types of selective autophagy have been identified, including mitophagy (Pickles et al., 2018), reticulophagy (ER-phagy) (Reggiori and Molinari, 2022), pexophagy (Germain and Kim, 2020), and xenophagy (Castaño‐Rodríguez et al., 2015).

ER-phagy specifically refers to the selective autophagic pathway responsible for the degradation of damaged endoplasmic reticulum (ER) and protein aggregates within the ER via lysosomes (Chen et al., 2020; Ferro-Novick et al., 2021). ER-phagy was first discovered in yeast by Bernales et al. in 2006, and the concept was formally proposed in 2007 (Bernales et al., 2006, 2007). ER-phagy relies on specific ER-phagy receptors, which both recognize and target damaged ER and protein aggregates, and interact with autophagy-related proteins to mediate the formation of autophagosomes. Through ER-phagy, the ER is fragmented and delivered to lysosomes for degradation and recycling (Bernales et al., 2007; Reggiori and Molinari, 2022). To date, six ER-phagy receptors have been identified in mammals: family with sequence similarity 134 member B (FAM134B) (Khaminets et al., 2015), secretory translocation protein (SEC62) (Fumagalli et al., 2016; Song et al., 2021), reticulon 3 long isoform (RTN3L) (Grumati et al., 2017), cell-cycle progression gene 1 (CCPG1) (Smith et al., 2018), atlastins 3 (ATL3) (Chen et al., 2019), and testis-expressed protein 264 (TEX264) (An et al., 2019).

ER-phagy plays an essential role in viral replication. For example, FAM134B inhibits the replication of Ebola viruses, Dengue viruses (DENV), and Zika viruses (ZIKV) (Chiramel et al., 2016; Lennemann and Coyne, 2017); SEC62-mediated ER-phagy suppresses Foot-and-mouth disease virus replication (Wu et al., 2021); and RTN3 binds to the amphipathic α-helical (AH) domain AH2 of Hepatitis C virus (HCV) NS4B, inhibiting its oligomerization and suppressing HCV replication (Wu et al., 2014). It is worth noting that ER-phagy receptors play distinct roles in different viral replication processes. Knockdown of RTN3 or ATL3 significantly reduces the egress of DENV and ZIKV, and West Nile virus (WNV) (Aktepe et al., 2017; Neufeldt et al., 2019); conversely, Severe Acute Respiratory Syndrome Coronavirus 2 (SARS-CoV-2) ORF3a activates FAM134B-dependent ER-phagy, thereby promoting viral replication (Zhang et al., 2022). On the other hand, viruses evolve strategies to counteract ER-phagy to ensure their replication and persistence (Chen et al., 2020; Li et al., 2021). For instance, DENV and ZIKV block the formation of viral proteins-containing autophagosomes (Lennemann and Coyne, 2017). A previous study indicates that PRRSV infection suppresses ER-phagy to facilitate viral replication (Guan et al., 2022). Additionally, our recent studies have shown that PRRSV Nsp5 plays a pivotal role in antagonizing innate immunity by activating FAM134B-mediated ER-phagy to degrade RLR signaling proteins (Wang et al., 2024). These findings highlight a complex ER-phagy regulatory network during PRRSV infection. Notably, our earlier findings were largely based on Nsp5 overexpression. Therefore, the functions of other PRRSV nonstructural proteins and the net effect of PRRSV whole-virus infection on ER-phagy require further investigation.

In this study, we demonstrate that during late infection stages, PRRSV employs its nonstructural proteins Nsp2 and Nsp5 to interact with and degrade FAM134B, thereby disrupting its interaction with LC3 and suppressing ER-phagy. Furthermore, we show that FAM134B exerts antiviral effects by restricting PRRSV replication, revealing a novel mechanism by which PRRSV counteracts host defenses through ER-phagy regulation. These findings provide critical insights into viral pathogenesis and highlight potential therapeutic targets for PRRSV control.

## 2 Materials and methods

### 2.1 Cell and viruses

HEK-293T and Marc-145 cell lines were obtained from the American Type Culture Collection (ATCC), and cultured in Dulbecco’s Modified Eagle Media (DMEM) (Solarbio, Beijing, China) supplemented with 10% Fetal Bovine Serum (FBS) (Gibco, San Diego, CA, USA), 100 IU/mL penicillin, and 100 μg/mL streptomycin (Solarbio, Beijing, China). The cells were maintained at 37°C in a humidified incubator with 5% CO_2_.

The PRRSV strain HN07-1 (GenBank accession no. KX766378.1) was stored at our laboratory.

### 2.2 Antibodies

Mouse anti-β-actin monoclonal antibody (mAb; catalog no. 3700), rabbit anti-HA mAb (catalog no. 3724) were purchased from Cell Signaling Technology (Danvers, MA, USA). Mouse anti-Flag mAb (catalog no. F3165) was purchased from Sigma-Aldrich (St. Louis, MO, USA). Rabbit anti-mCherry pAb (catalog no. 26765-1-AP) was purchased from Proteintech (Wuhan, China). Rabbit anti-PRRSV N protein pAb (catalog no. GTX129270) was purchased from Genetex (Irvine, CA, USA). HRP-goat anti-mouse IgG (catalog no. A21010) and HRP-goat anti-rabbit IgG (catalog no. A21010) secondary antibodies were purchased from Abbkine (Wuhan, China). Anti-DYKDDDDK magnetic agarose and Anti-HA magnetic beads were purchased from Invitrogen (Carlsbad, CA, USA).

### 2.3 Plasmids and transfection

The full-length PRRSV Nsps genes were amplified from the proviral cDNA extracted from HN07-1-infected Marc-145 cells and cloned into the PCAGGS-HA vector (stored at our laboratory) to generate recombinant PCAGGS-HA expression plasmids for Nsp1α, Nsp1β, Nsp2, Nsp3, Nsp4, Nsp5, Nsp6, Nsp7, Nsp8, Nsp9, Nsp10, Nsp11, and Nsp12. All primers were synthesized by Sangon Biotech (Shanghai, China). Plasmids were transfected using PL transfection reagent (TransGen Biotech, Beijing, China), following the manufacturer’s protocol, and 2.0 μg of plasmid DNA was used per well for six-well plates.

### 2.4 Western blot and immunoprecipitation

Treated cells were washed three times with ice-cold PBS to remove culture medium, and lysed on ice for 10 min using a cell lysis buffer suitable for both western blot and IP (Beyotime Biotechnology, Beijing, China). After centrifugation to remove cell debris, the lysates were incubated with HA or Flag conjugated magnetic beads for immunoprecipitation. The immunoprecipitated samples were then resolved by sodium dodecyl sulfate-polyacrylamide gel electrophoresis (SDS-PAGE) and transferred onto polyvinylidene fluoride (PVDF) membrane. To minimize nonspecific binding, the membranes were blocked with 5% non-fat milk for 1 h at room temperature, followed by overnight incubation with primary antibodies at 4°C. Unbound antibodies were removed by washing with TBST, and the membranes were subsequently incubated with HRP-conjugated secondary antibodies for 1 h at room temperature. Protein signals were visualized using an ECL chemiluminescent substrate (New Cell & Molecular) and detected with a chemiluminescence imaging system.

### 2.5 Quantitative RT-PCR

Total RNA was extracted using RNAiso Plus (Takara, catalog no. 9108), followed by reverse transcription using PrimeScript™ RT Master Mix (Takara, catalog no. RR036). Quantitative PCR was performed using SYBR Green relative PCR master mix (Vazyme, Nanjing, China) on a 7500 Fast instrument (Applied Biosystems, Foster, CA, USA), with glyceraldehyde-3-phosphate dehydrogenase (GAPDH) mRNA as the endogenous control. Relative gene expression levels were calculated using the 2^−△△Ct^ method. All primers were synthesized by Sangon Biotech.

### 2.6 TCID_50_ assay

The harvested virus samples were subjected to three freeze-thaw cycles, followed by clarification at 5,000 × g for 5 min. Viral titers were determined by TCID_50_ assays in Marc-145 cells using the Reed-Muench method. Briefly, tenfold serial dilutions (up to 10^-8^), of the virus stock were inoculated onto Marc-145 cell monolayers. After 7 days of incubation, cytopathic effect (CPE) was assessed to calculate the viral titer.

### 2.7 Statistical analysis

Differences between two groups were evaluated by the student’s t-test using GraphPad Prism (GraphPad Software, La Jolla, CA, USA). Differences were considered significant when p < 0.05 (^*^p < 0.05, ^**^p < 0.01).

## 3 Results

### 3.1 PRRSV inhibits ER-phagy during late infection

As previously reported, PRRSV inhibits ER-phagy (Guan et al., 2022). To further investigate the mechanisms underlying this suppression, we employed a reporter construct encoding mCherry-Sec61B. Sec61B is a resident protein of ER sheets. Upon ER-phagy, partial degradation of Sec61B liberates free mCherry, which can be detected by western blot as a marker of autophagic flux.

**Figure 1:**
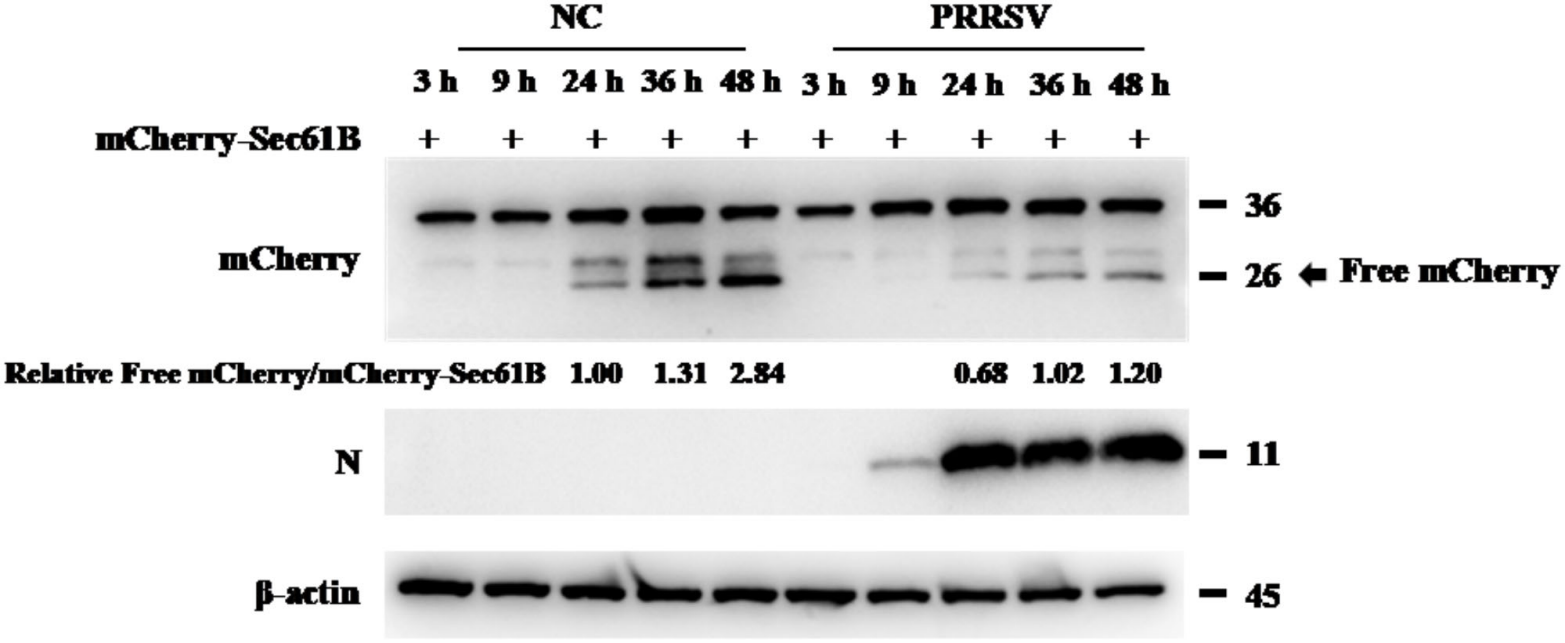
PRRSV suppresses ER-phagy during the late-stage infection. Marc-145 cells were transfected with mCherry-Sec61B for 24 h, followed by infection with PRRSV strain HN07–1 for 3, 9, 24, 36, or 48 h. The expression levels of mCherry and PRRSV N protein were analyzed by western blot. β-actin was used as a loading control.

Marc-145 cells were transfected with the mCherry-Sec61B plasmid using PL transfection reagent and subsequently infected with PRRSV or mock-infected. Samples were harvested at 3, 9, 24, 36, and 48 hours post-infection (hpi) for western blot analysis. As shown in **Figure 1**, free mCherry protein levels in the control group increased significantly by 24 hpi, indicating enhanced ER-phagy. In contrast, PRRSV-infected cells exhibited a marked reduction in relative free mCherry/mCherry-Sec61B levels at 24, 36, and 48 hpi compared to mock-infected controls. These results confirm that PRRSV suppresses ER-phagy during the late stages of PRRSV infection.

### 3.2 PRRSV degrades FAM134B and impairs its function

**Figure 2:**
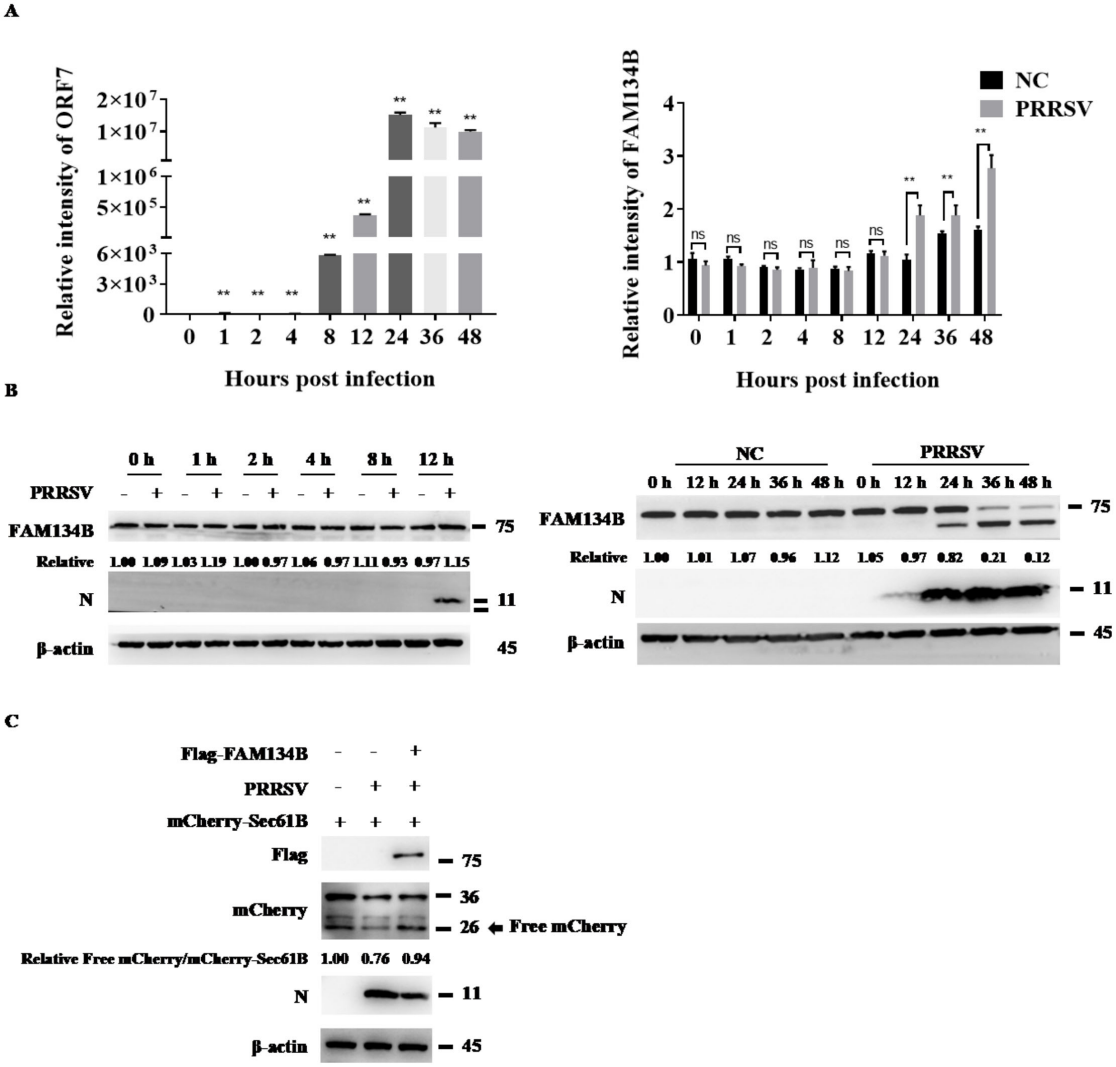
PRRSV induces FAM134B degradation. Marc-145 cells were infected with PRRSV strain HN07–1 for 0, 1, 2, 4, 8, 12, 24, 36 or 48 h. The mRNA levels of N and FAM134B **(A)** were detected by qRT-PCR. **(B)** The protein levels of FAM134B and N protein were analyzed by western blot. β-actin was set as an internal control. **(C)** FAM134B impedes PRRSV-mediated Sec61B degradation. Marc-145 cells were co-transfected with mCherry-Sec61B and Flag-FAM134B for 24 h, followed by infection with PRRSV strain HN07–1 for 24 h. The cell lysates were immunoblotted for mCherry-tag, Flag-tag (FAM134B), PRRSV N protein and β-actin.

To elucidate the molecular mechanism underlying the PRRSV-mediated inhibition of ER-phagy, we examined the transcription and protein levels of FAM134B, the first identified and key ER-phagy receptor (Khaminets et al., 2015). Marc-145 cells were either infected with PRRSV or mock-infected, and samples were collected at 0, 1, 2, 4, 8, 12, 24, 36 and 48 hpi for analysis using qRT-PCR and western blot. FAM134B mRNA levels are markedly upregulated at 24, 36, and 48 h post PRRSV infection compared to those in mock-infected cells (**Figure 2A**), indicating that viral infection promotes FAM134B transcription. However, endogenous FAM134B protein expression was significantly reduced at 24, 36, 48 hpi in Marc-145 cells (**Figure 2B**), consistent with the suppression of ER-phagy by PRRSV during late infection.

To verify whether FAM134B degradation contributes to ER-phagy suppression, Marc-145 cells were transfected with mCherry-Sec61B along with Flag-FAM134B or an empty vector, followed by infection with PRRSV strain HN07–1 or left untreated. As shown in **Figure 2C**, PRRSV infection reduced free mCherry release, indicating impaired ER-phagy flux. Importantly, this reduction was partially reversed by overexpression of FAM134B, suggesting that FAM134B degradation contributes to PRRSV-induced suppression of ER-phagy.

**Figure 3:**
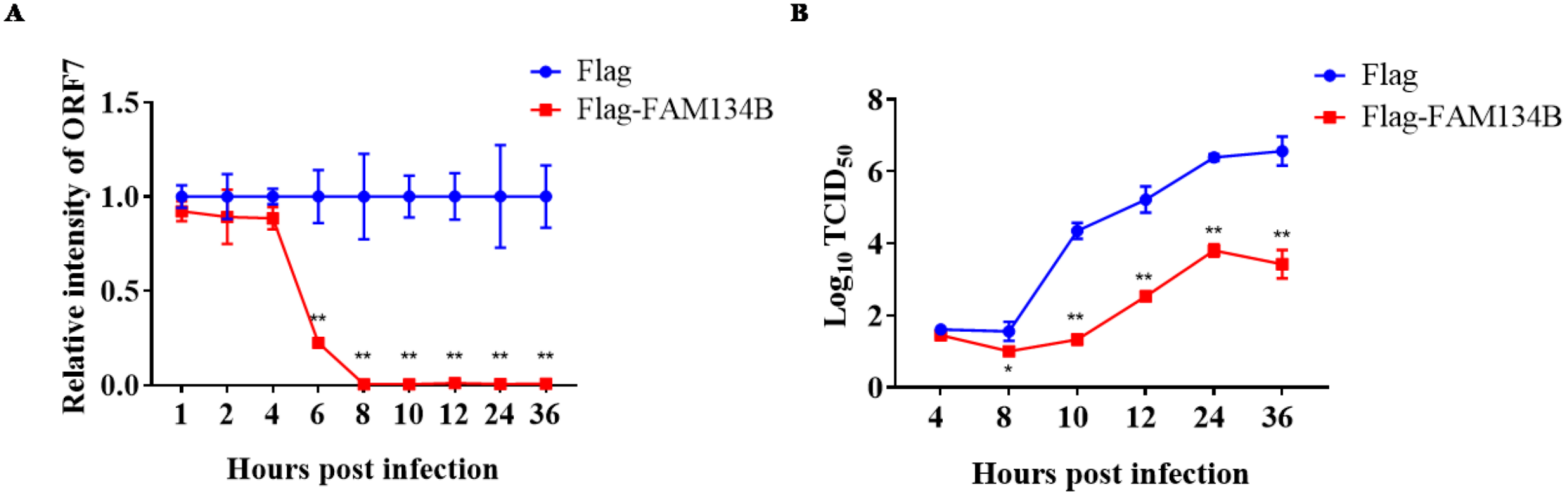
FAM134B inhibits PRRSV replication. **(A)** Time-course analysis of PRRSV ORF7 mRNA levels following FAM134B overexpression. Marc-145 cells were transfected with FAM134B (or empty Flag-vector as control) for 24 h, followed by PRRSV strain HN07–1 infection for 1, 2, 4, 6, 8, 10, 12, 24, or 36 h. The cell lysates were collected to detect ORF7 mRNA levels by qRT-PCR. Values are presented as fold changes relative to the Flag-control at each respective time point (value = 1. **(B)** FAM134B reduces PRRSV infectious titers. Marc-145 cells were transfected with FAM134B for 24 h, and then infected with PRRSV strain HN07–1 for 4, 8, 10, 12, 24, or 36 h. Supernatants were harvested after freeze-thaw cycles, and the viral titers were calculated using TCID_50_ assay.

To confirm the functional role of FAM134B in PRRSV infection, Marc-145 cells were transfected with exogenous FAM134B-expressing plasmid for 24 h, then infected with PRRSV HN07-1. qRT-PCR was performed at 1, 2, 4, 6, 8, 10, 12, 24, and 36 h post infection, and TCID₅₀ assays were performed at 4, 8, 10, 12, 24, and 36 h post infection. Both qRT-PCR and TCID_50_ assays consistently showed that FAM134B overexpression significantly reduced viral RNA abundance and extracellular progeny virus production (**Figure 3**). The antiviral effect became significant after 10 hpi. These findings indicate that FAM134B restricts PRRSV infection in Marc-145 cells.

### 3.3 PRRSV Nsp1α, Nsp1β, Nsp2, Nsp5, and Nsp11 reduce FAM134B protein levels

**Figure 4:**
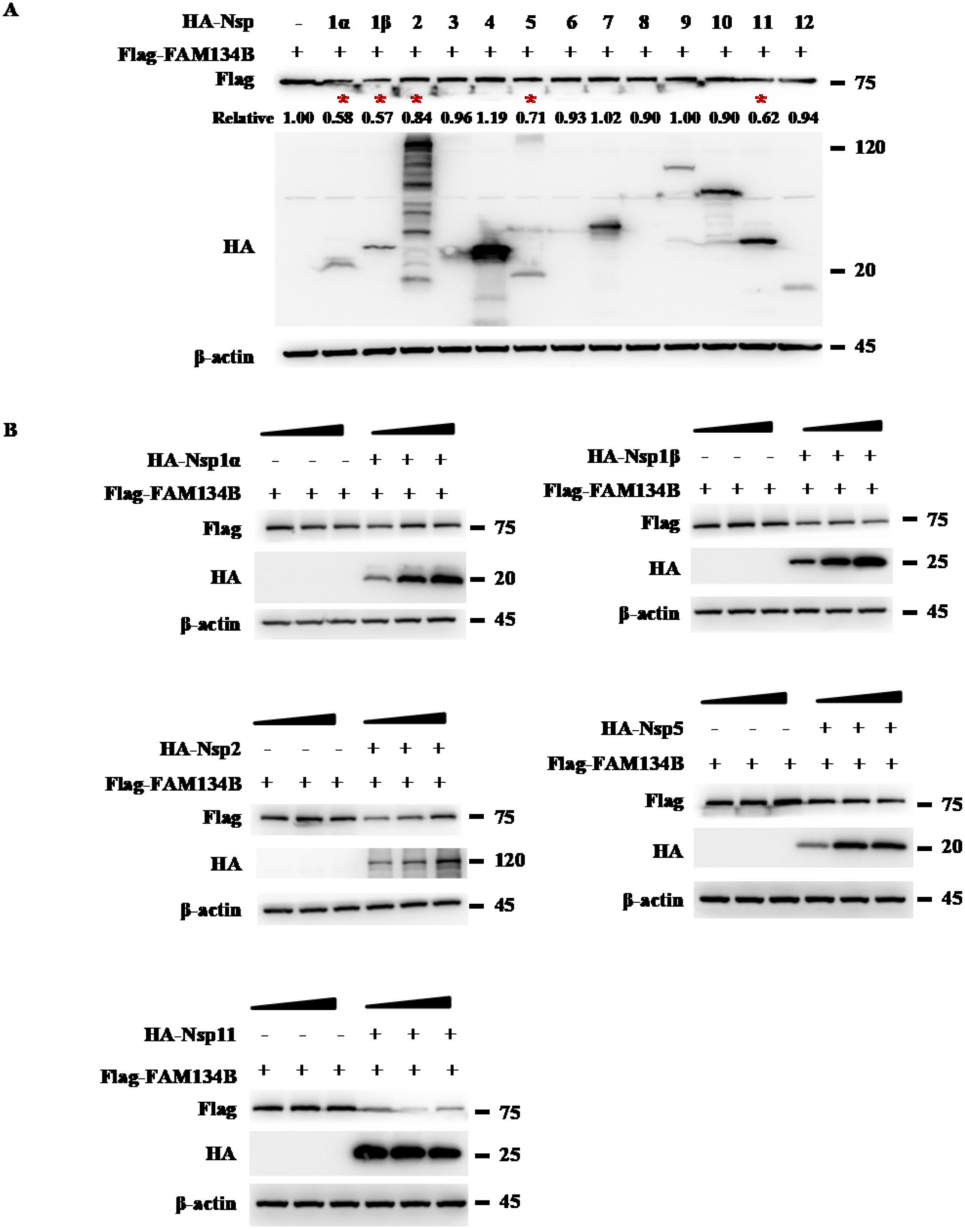
PRRSV Nsp1α, Nsp1β, Nsp2, Nsp5, and Nsp11 decrease FAM134B protein levels. **(A)** Screening of PRRSV Nsps regulating FAM134B expression. HEK-293T cells were co-transfected with HA-Nsp1α, Nsp1β, Nsp2, Nsp3, Nsp4, Nsp5, Nsp6, Nsp7, Nsp8, Nsp9, Nsp10, Nsp11 and Nsp12 or an empty vector, along with Flag-FAM134B. The cell lysates were detected by western blot using antibodies against the Flag-tag, HA-tag. β-actin was set as an internal control. **(B)** Validation of FAM134B-suppressing Nsps. HEK-293T cells were transfected with gradually increased doses of HA-labeled Nsp plasmids (Nsp1α, Nsp1β, Nsp2, Nsp5 and Nsp11), and an empty vector was used as the control. The same amount of Flag-FAM134B plasmid was applied to all groups to examine the dose-dependent effect of these Nsp proteins. Western blot was performed as in **(A)**.

After confirming that PRRSV infection decreases FAM134B protein levels, we systematically screened the viral nonstructural proteins for this activity. The plasmids encoding PRRSV HN07–1 Nsps were transfected into HEK293T cells to evaluate their effects on FAM134B protein levels. Expression of Nsp1α, Nsp1β, Nsp2, Nsp5, or Nsp11 significantly decreased FAM134B protein levels compared to the control group, whereas other Nsps showed no detectable effect (**Figure 4A**). To elucidate the regulatory mechanism, we conducted dose-response experiments, which demonstrated that these five Nsps promote FAM134B degradation (**Figure 4B**). These findings identify specific viral effectors responsible for PRRSV-mediated FAM134B degradation. We next investigated whether these Nsps directly interact with FAM134B.

### 3.4 FAM134B interacts with PRRSV Nsp2 and Nsp5 protein

**Figure 5:**
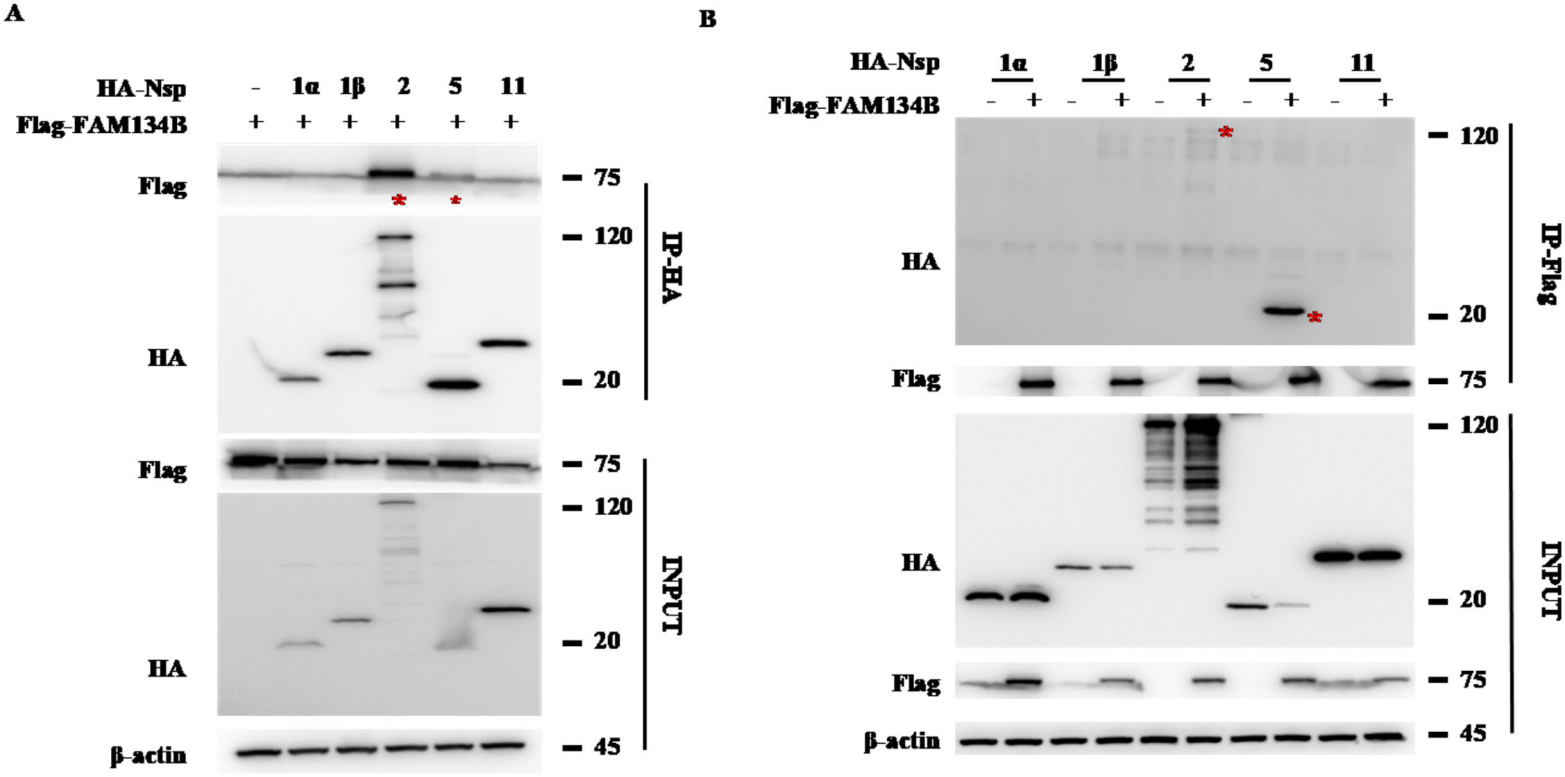
FAM134B interacts with PRRSV Nsp2 and Nsp5. **(A)** Co-IP analysis of FAM134B with PRRSV Nsps interactions. HEK-293T cells were co-transfected with HA-Nsp1α, Nsp1β, Nsp2, Nsp5, and Nsp11 or an empty vector, along with Flag-FAM134B. Then, the cell lysates were immunoprecipitated with HA-beads, and co-precipitated Flag-FAM134B was detected by anti-Flag western blot. **(B)** Reciprocal Co-IP confirming FAM134B-Nsp2/Nsp5 interaction. HEK-293T cells were co-transfected with Flag-FAM134B or an empty vector, along with HA-Nsp1α, Nsp1β, Nsp2, Nsp5, and Nsp11. Immunoprecipitation was performed using Flag-beads, and co-precipitated HA-Nsps were detected by anti-HA western blot.

Next, co-immunoprecipitation (Co-IP) was performed to assess the interaction between FAM134B and Nsp1α, Nsp1β, Nsp2, Nsp5 or Nsp11. HEK-293T cells were co-transfected with Flag-FAM134B together with HA-Nsp1α, Nsp1β, Nsp2, Nsp5 and Nsp11 plasmids or an empty vector for 24 h. The cell lysates were then immunoprecipitated using anti-HA or anti-Flag magnetic beads, and the bound proteins were analyzed by western blot. The results demonstrated that only Nsp2 and Nsp5 co-immunoprecipitated with FAM134B (**Figure 5**).

**Figure 6:**
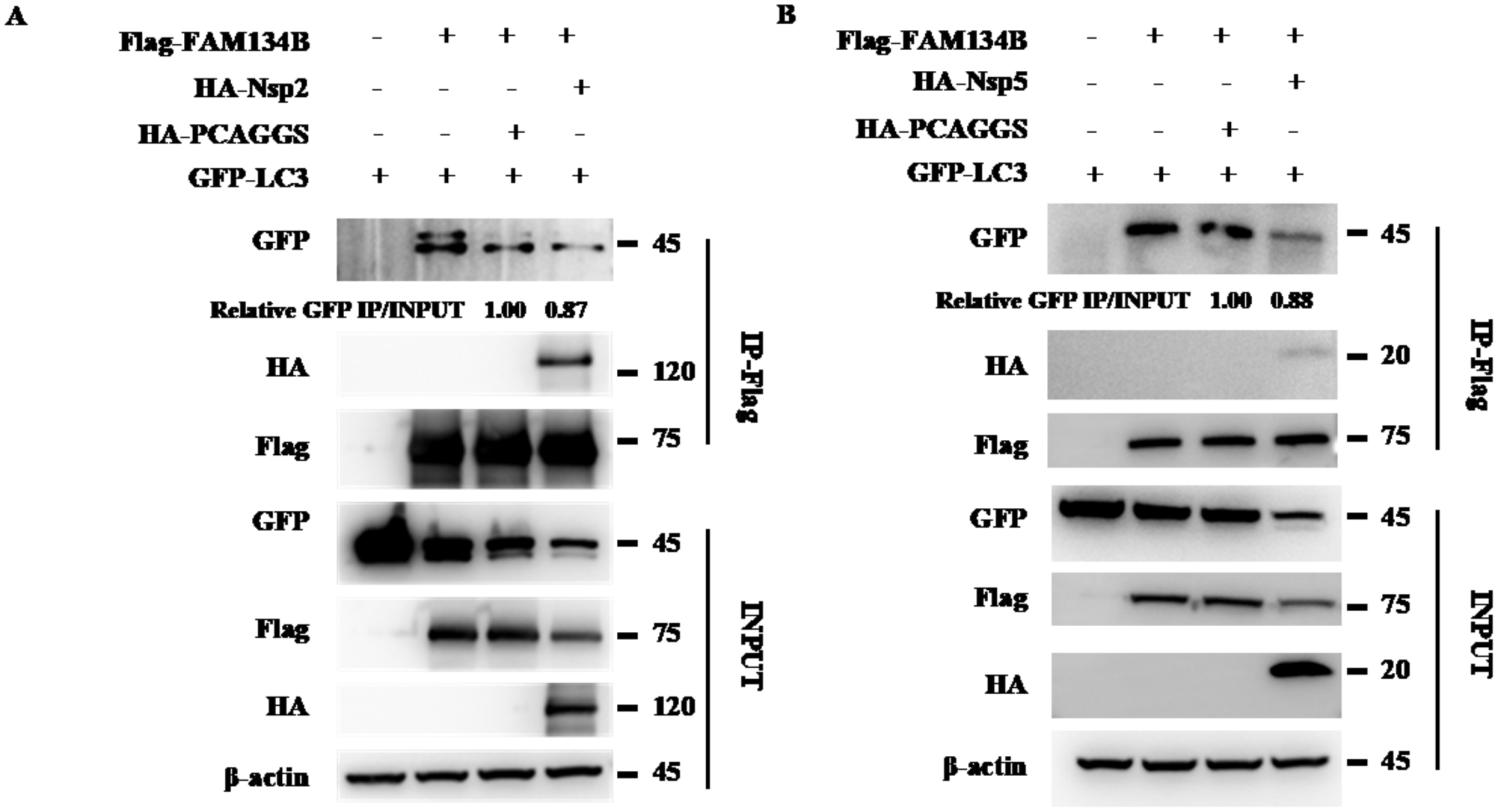
Nsp2 and Nsp5 disrupt the interaction between FAM134B and LC3. HEK-293T cells were co-transfected with GFP-LC3, Flag-FAM134B (or empty vector), and either HA-Nsp2 **(A)**, HA-Nsp5 **(B)** or an empty vector. At 24 hpt, the cell lysates were immunoprecipitated with Flag-beads. Co-precipitated GFP-LC3 and HA-Nsp2/5 were analyzed by western blot using anti-GFP and anti-HA antibodies, respectively.

Given that ER-phagy receptors typically function through LC3 binding for autophagosomal delivery of ER substrates (Birgisdottir et al., 2013; Johansen and Lamark, 2019), we examined whether Nsp2 or Nsp5 affect the FAM134B-LC3 interaction. These results showed that overexpression of Nsp2 or Nsp5 weakened the interaction between GFP-LC3 and FAM134B (**Figure 6**). Together with our earlier finding that Nsp2 and Nsp5 promote FAM134B degradation (**Figure 4**), these data indicate that Nsp2 and Nsp5 promote FAM134B degradation and disrupt its interaction with GFP-LC3, thereby suppressing PRRSV-induced ER-phagy.

## 4 Discussion

ER-phagy is a selective autophagy pathway that eliminates redundant ER (Bernales et al., 2006, 2007; Mochida and Nakatogawa, 2022). Previous work has shown that PRRSV suppresses ER-phagy through the inhibition of FAM134B expression, thereby promoting viral replication (Guan et al., 2022). In this study, we further elucidated the molecular mechanisms underlying PRRSV-induced ER-phagy suppression and FAM134B degradation. We found that FAM134B degradation begins at 24 hpi, coinciding with PRRSV-induced ER-phagy inhibition (**Figure 1**). Overexpression of Nsp1α, Nsp1β, Nsp2, Nsp5, and Nsp11 confirmed their role in FAM134B degradation (**Figure 4**). Notably, PRRSV Nsp2 and Nsp5 interacted with FAM134B (**Figure 5**), triggering FAM134B degradation and thereby disrupting ER-phagy. Collectively, these findings highlight the complex interplay between PRRSV infection and the ER-phagy pathways.

Autophagy is classified as complete or incomplete autophagy based on whether autophagosomes fuse with lysosomes. When fusion-blocking inhibitors are used, PRRSV infection does not alter LC3-I to LC3-II conversion (Sun et al., 2012; Zhou et al., 2023). Additionally, confocal microscopy has revealed that LC3 fails to colocalize with the lysosomal marker LAMP2 post-infection (Zhang et al., 2019), indicating that PRRSV infection induces incomplete autophagy. However, with prolonged PRRSV infection, P62 protein expression decreases in a time-dependent manner (Liu et al., 2012; Zhou et al., 2016), suggesting a shift toward complete autophagy at later time points. These findings suggest that PRRSV dynamically regulates autophagy pathways in a phase-dependent manner. Consistent with this notion, a recent study from our group further revealed that PRRSV Nsp5 induces FAM134B-mediated ER-phagy to suppress innate immunity by degrading RLR signaling proteins (Wang et al., 2024). That study also identified the Nsp5 86–122 aa region as the FAM134B-binding domain; however, PRRSV mutants lacking this region were non-viable, precluding functional validation of this interaction in the context of viral infection. The present study using PRRSV whole-virus infection reveals that ER-phagy is suppressed during late infection (**Figure 1**). The increase in FAM134B mRNA upon PRRSV infection, despite reduced protein levels, likely reflects a cellular compensatory response to counteract virus-induced protein degradation, although the underlying mechanism remains to be elucidated. These findings collectively raise the interesting possibility that PRRSV may regulate ER-phagy in a time-dependent manner, with activation at early stage followed by suppression at late stage to preserve ER integrity for viral replication, a hypothesis that warrants further investigation. Additionally, the precise degradation pathway responsible for Nsp2/Nsp5-mediated FAM134B turnover remains to be determined. Both Nsp2 and Nsp5 have been reported to engage both degradation systems to eliminate distinct host substrates (Diao et al., 2023; Huang et al., 2025; Li et al., 2024; Wang et al., 2024; Yang et al., 2017; Zhao et al., 2024; Zheng et al., 2026; Zhu et al., 2025, 2023). Beyond the Nsp2/Nsp5-mediated degradation, Nsp1α, Nsp1β, and Nsp11 also reduced FAM134B protein levels, Co-IP assays detected no direct interaction between these Nsps and FAM134B (**Figure 5**), suggesting that they may downregulate FAM134B through indirect mechanisms. Elucidating these pathways warrants further investigation. Collectively, these findings provide new directions for future research, including whether PRRSV targets other ER-phagy receptors and how the dynamic balance between ER-phagy activation and suppression is regulated during infection to maintain immunity and ER homeostasis.

ER-phagy is generally accompanied by ER stress (ERS) (Ferro-Novick et al., 2021; He and Klionsky, 2009; Sun and Brodsky, 2019). During SARS-CoV-2 infection, ORF3a induces FAM134B-dependent ER-phagy, triggering subsequent ER stress and inflammatory responses (Zhang et al., 2022). Similarly, SARS-CoV-2 Nsp6 mediates the host innate immune response by degrading STING via ERS and ER-phagy (Jiao et al., 2023). Similar to SARS-CoV-2, PRRSV also manipulates ER-phagy and ERS to modulate host cellular responses. Previous studies have shown that PRRSV Nsp2 and Nsp5 induce ERS (Diao et al., 2023). Here, we found that PRRSV suppresses ER-phagy by degrading FAM134B via Nsp2 and Nsp5 (**Figure 4**). Therefore, further research is needed to understand how PRRSV modulates ERS and ER-phagy, as well as the precise functions of Nsp2 and Nsp5. Nsp2 and Nsp5 are involved in the formation and maintenance of double-membrane vesicles (DMV) during PRRSV infection (Pedersen et al., 1999), thereby facilitating viral RNA synthesis and replication. Notably, both PRRSV-induced DMVs (Zhang et al., 2018) and Nsp5-triggered autophagosomes originate from the ER (Wang et al., 2024; Zhang et al., 2019), highlighting the critical role of the ER in viral infection and autophagy processes. The interaction between viral proteins/replication complexes and ER membranes drives ER expansion and remodeling to facilitate DMV formation. This suggests that Nsp5-induced ER localization may not only facilitate DMV formation but also modulate the assembly and function of viral and host proteins by regulating protein folding and modification.

In conclusion, this study demonstrates that during late stage of infection, PRRSV employs Nsp2 and Nsp5 to interact with and degrade FAM134B, thereby disrupting its interaction with LC3 and suppressing ER-phagy. These findings not only confirm the inhibitory role of ER-phagy and FAM134B in PRRSV replication but also reveal a novel mechanism by which PRRSV modulates ER-phagy to facilitate viral replication.

## Data Availability

The original contributions presented in the study are included in the article/supplementary material. Further inquiries can be directed to the corresponding authors.
